# OCT angiography indices and the choroidal vascularity index in wild-type transthyretin (TTR) amyloidosis (ATTRwt)

**DOI:** 10.3389/fmed.2023.1174643

**Published:** 2024-01-15

**Authors:** Michele Rinaldi, Fausto Tranfa, Flavia Chiosi, Giuseppe Campagna, Maddalena De Bernardo, Marco Gioia, Francesco Natale, Martina Caiazza, Francesca Dongiglio, Federica Verrillo, Giuseppe Palmiero, Giuseppe Limongelli, Ciro Costagliola

**Affiliations:** ^1^Department of Ophthalmology, Università degli studi della Campania “Luigi Vanvitelli”, Naples, Italy; ^2^Department of Neuroscience, Reproductive and Dentistry Sciences, Federico II University of Naples, Naples, Italy; ^3^Department of Ophthalmology, Azienda Ospedaliera dei Colli, Monaldi Hospital, Naples, Italy; ^4^Department of Medical-Surgical Sciences and Translational Medicine, University of Rome “Sapienza”, Rome, Italy; ^5^Eye Unit, Department of Medicine Surgery and Dentistry “Scuola Medica Salernitana”, University of Salerno, Baronissi, Italy; ^6^Department of Cardiothoracic Sciences, Università degli studi della Campania “Luigi Vanvitelli”, Monaldi Hospital, Naples, Italy; ^7^Inherited and Rare Cardiovascular Diseases, Department of Translational Medical Sciences, University of Campania “Luigi Vanvitelli”, Monaldi Hospital, Naples, Italy

**Keywords:** choroidal vascularity index (CVI), retinal and choroidal microangiopathy, retinal vessel density, wild-type (ATTRwt) transthyretin amyloidosis, OCT angiography (OCTA)

## Abstract

**Purpose:**

Retinal angiopathy represents a well-known ocular manifestation of hereditary transthyretin amyloidosis (ATTRv). Until recently, there have been no reports on retinal changes in ATTRwt. In this retrospective observational clinical study, we aimed to determine whether vessel density (VD) indices and the choroidal vascularity index (CVI) could offer insights into retinal and choroidal vascular changes among patients affected by ATTRwt.

**Methods:**

Eighteen patients with a confirmed diagnosis of ATTRwt underwent structural optical coherence tomography (OCT) and OCT angiography (OCTA). We established a control group consisting of 16 healthy subjects for statistical comparisons. The 3D OCT and OCTA datasets were analyzed to assess retinal and choroidal thickness and VD. For measuring CVI, we obtained measurements for the total choroid area (TCA), luminal area (LA), and stromal area (SA).

**Results:**

The mean VD exhibited a statistically significant reduction in the superficial capillary plexus (SCP), deep capillary plexus (DCP), and choriocapillaris (CC) among the ATTRwt group in comparison to the control group (*p* < 0.0001). Notably, ATTRwt patients displayed decreased choroidal thickness (*p* = 0.08). Additionally, the median CVI was lower in the ATTRwt group than in the control group (*p* = 0.04).

**Conclusion:**

The indices from OCTA and CVI have the potential to serve as non-invasive biomarkers for the quantitative evaluation of retinal and choroidal vascular involvement in patients with ATTRwt.

## Introduction

Systemic amyloidosis represents a collection of rare diseases characterized by the multiorgan deposition of amyloid fibrils, a substance formed from the misfolding of proteins (1). In transthyretin amyloidosis, systemic symptoms arise from breaking the tetrameric structure into monomers of the transthyretin (TTR) plasma protein (2). These monomers are inclined to aggregate into amyloid fibrils within the extracellular space of surrounding tissues, leading to damage that affects organ function.

Transthyretin amyloidosis (ATTR) encompasses two distinct pathologies based on whether the amyloid fibrils originate from the intact molecule of TTR: wild-type ATTR (ATTRwt), also known as senile systemic amyloidosis, or from mutations occurring in the TTR gene (ATTRv) (3).

TTR is a carrier for thyroxine (T4) and retinol (vitamin A). It is primarily produced by the liver, brain choroid plexus, and retinal pigment epithelium, thereby playing a role in vitreoretinal metabolism and the visual cycle (4). Consequently, the deposition of TTR at the ocular level can occur through the local production of TTR within the eye that does not cross the Bruch's membrane and the circulating TTR coming into contact with the choroid (5). Recent attention has been directed toward choroidal vascular involvement in individuals affected by hereditary transthyretin amyloidosis using multimodal imaging techniques (5, 6). Choroidal amyloid angiopathy has been observed to be more prevalent than retinal amyloid angiopathy, with its severity being correlated with the systemic severity score (7, 8). These studies conducted their analyses based on evidence obtained from indocyanine green angiography, enhanced depth imaging (EDI), optical coherence tomography (OCT), and the calculation of the choroidal vascularity index (CVI). The most comprehensive study was conducted by Marta et al. who reported reduced choroidal thickness and CVI in 166 patients affected by ATTRv compared to a healthy population (5).

In patients with systemic diseases, qualitative and quantitative abnormalities of retinal and choroidal perfusion can be assessed by detecting vessel densities (VD) within the superficial, deep, and choriocapillary plexuses through non-invasive analysis using OCT angiography (OCTA). Hereditary transthyretin amyloidosis can significantly impact the eye, negatively affecting visual function. Furthermore, it is crucial to note that ocular involvement is not uniform and tends to vary among individual patients (9).

Marques et al. examined retinal angiopathy in individuals with hereditary transthyretin amyloidosis, indicating that patients with a scalloped iris were linked to a more advanced subclinical retinal angiopathy (10, 11).

This study aimed to assess whether VD indices and the choroidal vascularity index (CVI) offer a comprehensive analysis of the retinal and choroidal vascular conditions in individuals affected by ATTRwt compared to a healthy population.

## Materials and methods

This is an observational, retrospective, and case-control study conducted in the Department of Ophthalmology at Azienda Ospedaliera dei Colli Monaldi Hospital and in the Department of Inherited and Rare Cardiovascular Diseases at the University of Campania “Luigi Vanvitelli,” Monaldi Hospital of Napoli, spanning from January 2021 to September 2022. The study adhered to the principles of the Declaration of Helsinki. Written informed consent was obtained from individuals for the potential publication of identifiable images or data included in this article. The study included 20 patients with a confirmed diagnosis of ATTRwt (Group 1), and a healthy age-matched control group of 16 patients (Group 2) was also enrolled in the study.

All clinical data about clinical symptoms and characteristics associated with systemic amyloidosis were extracted from patient records from the Department of Inherited and Rare Cardiovascular Diseases at the University of Campania “Luigi Vanvitelli,” Monaldi Hospital.

A comprehensive eye examination was conducted for all patients, including swept-source OCT (SS-OCT) and OCTA.

The inclusion criteria for Group 1 comprised a confirmed diagnosis of ATTRwt, validated through genetic testing, the presence of signs or symptoms, and an age >18 years. Group 2 consisted of healthy subjects matched in age to the study group.

Exclusion criteria for both groups encompassed the presence of comorbidities such as diabetes, atherosclerotic vasculopathy, glaucoma, other macular or retinal disorders, opacities in the optical media that could impede accurate retinal functional assessments, and the inability of patients to maintain visual fixation.

Demographic characteristics of the patients, including gender, age, age at diagnosis, months of follow-up since diagnosis, therapy (tafamidis), and systemic involvement (such as the presence of polyneuropathy, cardiomyopathy, or gastrointestinal disorders), were recorded.

The ophthalmic assessment included the determination of best-corrected visual acuity (BCVA) using Early Treatment Diabetic Retinopathy Study (ETDRS) charts, slit-lamp examination, intraocular pressure measurement, and fundus examination after pupil dilation. Central foveal thickness (CFT) and choroidal thickness (CT) were quantified using swept-source OCT (SS-OCT) in both study groups.

Within Group 1, two patients exhibited retinal alterations at the posterior pole and were subsequently excluded from the study.

### Scanning protocol and quantitative analysis of OCT and OCTA images

All images were captured after pupil dilation using 1.0% tropicamide eyedrops. Analysis of SS-OCT and OCTA data was carried out to ascertain retinal and choroidal thickness, vessel density (VD), and CVI employing the swept-source Topcon DRI OCT Triton (Topcon Corporation, Japan). Among all subjects, only the eye with the highest image quality was employed for the analysis. The acquisition protocol encompassed a macular line scan and a 6 × 6 mm macular cube scan for both OCT and OCTA. Each scan was centered on the fovea, and the IMAGENET 6.0 automated layer detection tool was employed to delineate retinal layers for the assessment of the superficial capillary plexus (SCP), deep capillary plexus (DCP), and choriocapillaris (CC). Three-dimensional datasets were scrutinized to define retinal thickness and VD within the five ETDRS sectors (central, nasal, temporal, inferior, and superior).

Only images with a quality score >50 were deemed acceptable and subsequently included in the final analysis. VD was determined as the proportion of the total area covered by blood vessels. When utilizing OCTA on the Topcon DRI OCT Triton, the unit of measurement is typically expressed as a dimensionless ratio or percentage. This metric quantifies the concentration of blood vessels within a specific region of interest in the scanned tissue, representing the relative extent of the analyzed area occupied by blood vessels. This unit of measurement aligns consistently with the established method for characterizing vessel density in OCTA imaging across different devices and platforms.

The OCTA software generated vessel densities for each scan within the identical ETDRS regions outlined in our study protocol. The OCTA signal was identified and recorded for every ETDRS region, overlaid onto each OCTA en face layer. Subsequently, the average normalized VD of the full-thickness retina and the choriocapillaris in each ETDRS region was extrapolated from every subject, enabling further analysis.

The CVI was measured using ImageJ software version 1.52. Specifically, the choroidal area was demarcated using the polygon tool and integrated into the ROI manager. The upper boundary of the evaluated area aligned with the RPE-Bruch's membrane complex, while the lower boundary corresponded to the choroidal-scleral junction. The nasal and temporal margins were aligned with the choroidal image's respective edges. Niblack's autolocal threshold technique was employed to transform grayscale images into binarized images. The image was then converted to RGB color, and the color threshold tool was applied to identify dark pixels (12, 13). The Total Choroidal Area (TCA) measurement encompassed the entire selected region, while the Luminal Choroidal Area (LCA) was derived from the area occupied by dark pixels. Furthermore, the stromal choroidal area (SCA) and the CVI were computed by subtracting LCA from TCA and calculating the ratio of LCA to TCA, respectively.

## Outcome measures

The main outcome we focused on was discerning variations in VD or the CVI between the two groups. Secondary outcome measures encompassed the CFT and CT.

## Statistical analysis

Continuous variables assessed in the SCPs, DCPs, choriocapillaris (CC), TCA, LCA, SCA, CVI, and age were presented as mean ± standard deviation (SD) along with their corresponding 95% confidence interval (95% CI), or as median with the range from minimum to maximum values. Categorical variables were displayed as absolute frequencies and percentages: *n* (%). The relationship between the ATTRwt and control groups regarding sex was assessed using the chi-squared (χ^2^) test. The normality of continuous variables was assessed using the Shapiro-Wilk test, QQ plot, and examination of kurtosis and skewness indices. To compare continuous variables between the ATTRwt and control groups, Student's *t*-test and the Brunner-Munzel (BM) test were employed. The BM test is preferred over the Mann-Whitney test when dealing with tied and skewed data, providing greater robustness (14–17).

All statistical analyses were conducted using SAS version 9.4 and JMP PRO version 17, developed by SAS Institute Inc. in Cary, NC.

## Results

Demographic and anamnestic data for all participants are presented in Table 1. No significant differences were observed in age and sex between Group 1 and Group 2 (*p* = 0.11 and *p* = 0.37, respectively). The mean age for Group 1 was 71.33 ± 6.62 (95% CI: 68.04–74.63), and for Group 2, it was 68.81 ± 9.01 (95% CI: 64.01–73.62). Regarding sex distribution, the proportions for Group 1 were female/male: 4 (22.22%)/14 (77.78%), and for Group 2, they were female/male: 6 (37.50%)/10 (62.50%).

**Table 1:** Demographic, genetic, and clinical characteristics in the ATTRwt group.

**Patients**	**Sex**	**Age years**	**Age at diagnosis**	**Follow-up months**	**TTR genetic test**	**Systemic involvement**			**Treatment (months since starting)**
						**PG**	**GI**	**CM**	
1	Male	75	74	10	WT	No	Yes	No	Tafamidis 61 (7)
2	Male	73	72	9	WT	Yes	Yes	No	Tafamidis 61 (5)
3	Male	69	69	3	WT	No	Yes	No	No
4	Male	65	65	5	WT	Yes	Yes	Yes	Tafamidis 61 (2)
5	Male	86	85	11	WT	Yes	Yes	No	Tafamidis 61 (6)
6	Male	71	71	3	WT	Yes	Yes	No	No
7	Male	68	68	5	WT	Yes	Yes	Yes	Tafamidis 61 (2)
8	Female	65	64	10	WT	Yes	Yes	Yes	Tafamidis 61(7)
9	Male	70	70	4	WT	No	Yes	No	No
10	Male	68	68	3	WT	No	Yes	No	Tafamidis 61(1)
11	Female	61	61	6	WT	Yes	Yes	No	Tafamidis 61(6)
12	Male	78	77	8	WT	No	Yes	No	Tafamidis 61 (5)
13	Male	76	75	7	WT	No	Yes	No	No
14	Female	78	77	9	WT	Yes	Yes	No	Tafamidis 61 (2)
15	Male	81	80	10	WT	No	Yes	No	Tafamidis 61 (3)
16	Male	65	64	6	WT	Yes	Yes	No	Tafamidis 61 (3)
17	Male	64	64	3	WT	No	Yes	No	No
18	Female	71	71	4	WT	No	Yes	No	No

TTR, transthyretin; WT, wild type; PN, polyneuropathy; GI, gastrointestinal involvement; CM, cardiomyopathy.

In Group 1, the mean ± SD age at the time of diagnosis was 70.83 ± 6.33 years, and the mean ± SD follow-up duration was 6.44 ± 2.85 months. Genetic testing ruled out the presence of an inherited form of amyloidosis, thereby confirming a diagnosis of wild-type ATTR. All subjects in this group were affected by transthyretin amyloid cardiomyopathy, with 50% reporting polyneuropathy and 38.8% reporting gastrointestinal disturbances. Among the participants, 12 patients were undergoing tafamidis therapy, a specific stabilizer of the TTR protein, with an average treatment duration of 4.08 ± 1.86 months. The remaining six patients did not receive any treatment. There was no statistically significant difference in BCVA between the two groups.

The intraocular pressure (IOP) was within the normal range for all patients, with a mean IOP of 14 mmHg. None of the examined subjects had been diagnosed with glaucoma.

### Swept-source optical coherence tomography angiography indices and choroidal vascularity index results

**Figure 1:**
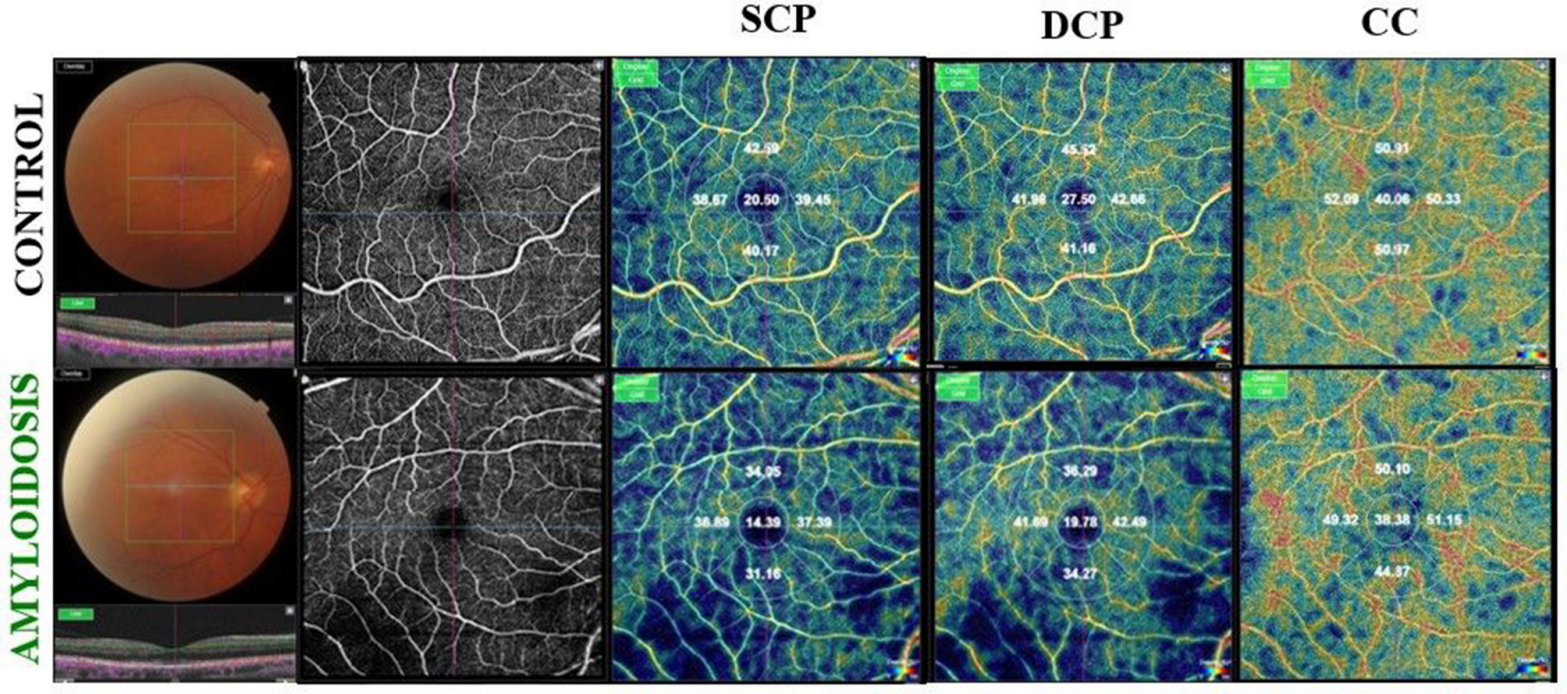
**(Upper)** Right eye of an healthy 67-year-old male shows on Optical Coherence Tomography Angiography (OCTA), a normal vascular texture of the superficial capillary plexus (SCP), the deep capillary plexus (DCP), and choriocapillaris (CC). **(Bottom)** On OCTA the right eye of an ATTRwt patient (71-year-old male) reveals a reduced foveal vessel density in SCP, DCP, and CC indices.

The mean VD, measured as a percentage (%), was significantly reduced in the SCP, DCP, and choriocapillaris (CC) across all five ETDRS sectors in Group 1 compared to the control group (*p* < 0.0001; Table 2, Figure 1).

**Table 2:** Differences in the vessel density indices between the control and amyloidosis groups.

	**Superficial capillary plexus**			**Deep capillary plexus**			**Choriocapillaris**		
**Parameter**	**Control group**	**Amyloidosis group**	***p***	**Control group**	**Amyloidosis group**	***p***	**Control group**	**Amyloidosis group**	***p***
	**Mean ±SD**	**Mean ±SD**		**Mean ±SD**	**Mean ±SD**		**Mean ±SD**	**Mean ±SD**	
	**(95% CI)**	**(95% CI)**		**(95% CI)**	**(95% CI)**		**(95% CI)**	**(95% CI)**	
Central mVD (%)	19.41 ± 1.61 (18.54–20.27)	15.66 ± 3.59 (13.87–17.45)	**<0.0001**	28.45 ± 2.68 (27.02–29.88)	23.45 ± 4.72 (21.10–25.80)	**0.0007**	45.71 ± 5.08 (40.71–48.12)	25.67 ± 4.33 (23.52–27.82)	**0.0005**
Superior^*^ (%)	42.12 ± 3.67 (40.17–44.08)	32.18 ± 7.16 (28.62–35.74)	**<0.0001**	43.98 (37.43–49.26)	32.59 (17.05–45.49)	**<0.0001**	49.46 (44.47–52.49)	40.66 (17.19–48.00)	**<0.0001**
Inferior^*^ (%)	43.24 ± 4.11 (41.05–45.43)	31.85 ± 7.51 (28.12–35.59)	**<0.0001**	44.36 ± 3.36 (42.57–46.15)	31.21 ± 9.57 (26.45–35.97)	**<0.0001**	51.38 (48.16–56.46)	42.23 (16.48–51.02)	**<0.0001**
Nasal^*^ (%)	40.43 ± 3.45 (38.59–42.47)	33.55 ± 5.34 (30.90–36.21)	**<0.0001**	43.42 ± 2.95 (41.85–44.99)	33.74 ± 6.86 (30.33–37.15)	**<0.0001**	50.06 (45.40–55.21)	42.88 (18.02–51.12)	**<0.0001**
Temporal^*^ (%)	40.70 ± 2.64 (39.29–42.11)	33.13 ± 7.81 (29.25–37.02)	**<0.0001**	43.92 ± 2.30 (42.70–45.14)	33.58 ± 8.33 (29.44–37.72)	**<0.0001**	50.38 (46.73–54.30)	42.02 (17.80–52.02)	**0.0009**

^*^Data presented as median (min to max).

^*^The comparisons were performed using the Brunner-Munzel test. Bold values indicate *p* value is statistically significant.

The mean CVI was significantly lower in the eyes with wild-type ATTR (ATTRwt) compared to the control group (*p* = 0.04) (Table 3).

**Table 3:** Comparison of the choroidal vascularity index between the control and amyloidosis groups.

**Parameter**	**Control group**	**Amyloidosis group**	***p***
	**Median (min to max)**	**Median (min to max)**	
TCA^*^	17.29 (7.98–21.81)	12.30 (4.28–17.91)	0.002
LCA^*^	7.69 (2.15–13.16)	4.35 (1.35–12.06)	0.003
SCA^*^	8.85 (3.95–12.07)	7.05 (2.43–10.69)	0.009
CVI^*^	41.57 (22.90–62.95)	32.86 (21.36–70.22)	0.08

^*^Comparisons were performed using the Brunner-Munzel test.

TCA, total choroid area; LCA, luminal choroid area; SCA, stromal choroid area; CVI, choroidal vascularity index.

CFT in micrometers (μm) did not show a statistically significant difference between the two groups. However, a trend toward reduced CT in micrometers (μm) was observed in ATTRwt patients (*p* = 0.08) compared to healthy subjects, although this difference was not statistically detectable.

Furthermore, the LCA and SCA were both found to be statistically significantly reduced in the amyloidosis group compared to the control group (*p* = 0.003 and *p* = 0.009, respectively) (Table 3).

**Figure 2:**
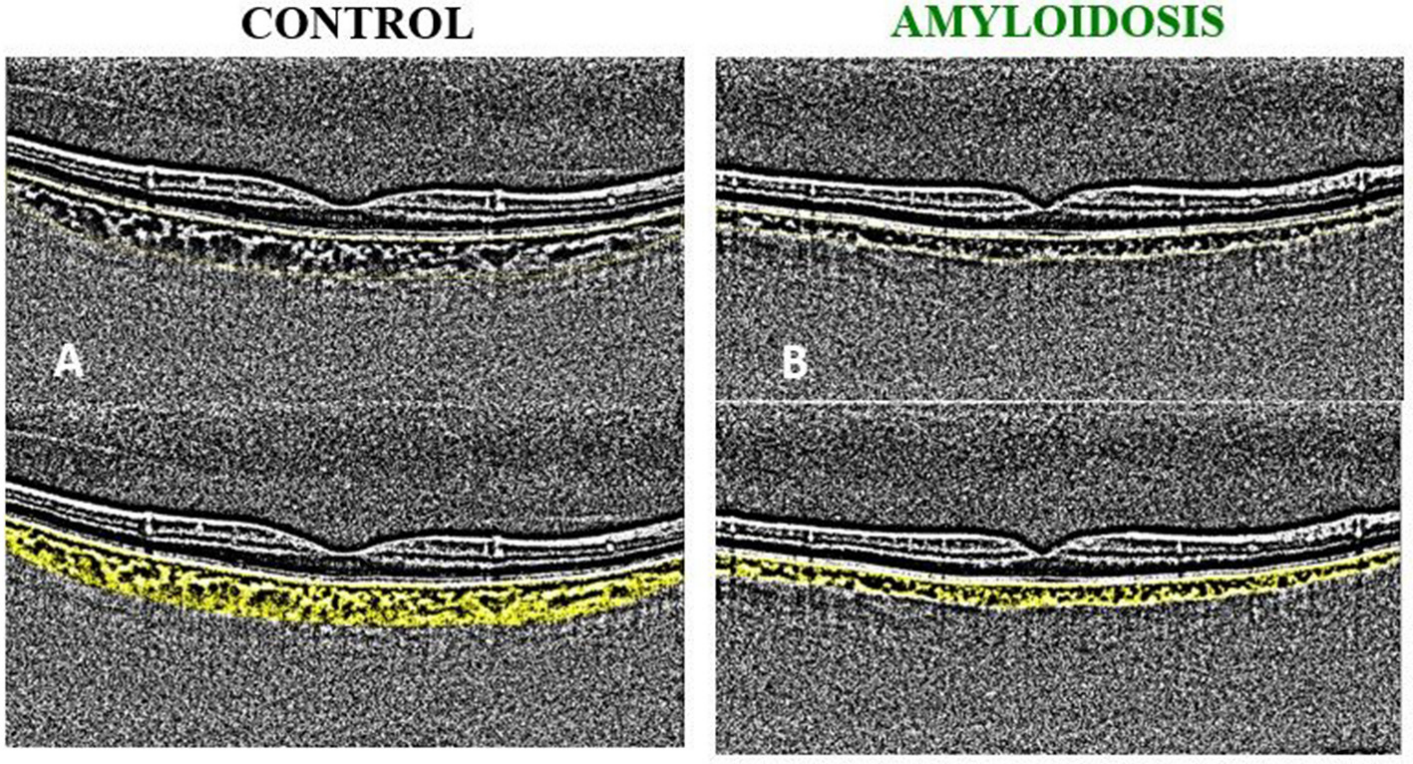
Example of binarized images for the calculation of choroidal vascularity index (CVI). **(A)** A 74 y/o healthy male: CVI = 34.01; TCA = 16.20; LCA = 5.51; SCA=10.69. **(B)** A 74 y/o ATTRwt male patient: CVI = 32.66; TCA = 7.47; LCA = 2.44; SCA = 5.03.

Figure 2 illustrates a representative binarized image depicting the choroidal status of an ATTRwt patient alongside a healthy subject of the same age and gender.

### Subgroup analysis

Within the ATTRwt group, a sub-analysis was performed based on tafamidis therapy. A comparison was conducted between patients receiving the therapy and those not undergoing treatment. No significant differences were observed regarding VD within any plexus or the CVI. The distribution of data remained consistent between the two groups.

### Ocular anterior and posterior segment findings

The ocular observations about the conjunctiva, cornea, lens, vitreous, and retina, primarily related to fibril deposition, are summarized in Table 4. Among the patients, 10 (55.5%) exhibited bilateral conjunctival ectasia, 6 (33.3%) showed bilateral corneal amyloid deposition, and four eyes had undergone pseudophakic procedures. Additionally, four patients' lenses had amyloid fibril deposition (22.2%). Notably, this population did not exhibit scalloped pupils or iris amyloid deposition.

**Table 4:** Anterior and posterior ocular findings in the amyloidosis group.

**Patients**	**Conjunctival ectasia**	**Corneal deposition**	**Lens deposition**	**Vitreous opacities**	**Retinal deposits**
1	Yes	No	Pseudophakia	Yes	No
2	No	Yes	Yes	Yes	No
3	Yes	No	No	Yes	No
4	Yes	No	No	Yes	No
5	No	Yes	Pseudophakia	No	No
6	Yes	No	Yes	Yes	No
7	No	No	No	No	No
8	No	Yes	No	No	No
9	Yes	No	Yes	Yes	No
10	No	No	No	No	No
11	Yes	No	Yes	Yes	No
12	Yes	Yes	Pseudophakia	Yes	No
13	Yes	Yes	Yes	Yes	No
14	No	No	No	Yes	No
15	No	No	Pseudophakia	No	No
16	Yes	No	Yes	Yes	No
17	No	Yes	No	No	No
18	Yes	No	No	Yes	No

Concerning the posterior segment, bilateral vitreous opacities were observed in 12 patients (66.6%), while no evidence of retinal deposits was found.

## Discussion

Ocular involvement in hereditary transthyretin amyloidosis has been described with numerous variants and frequent asymmetrical manifestations. Reported findings include the deposition of amyloid fibrils at the anterior capsule of the crystalline lens and the pupillary border (resulting in a scalloped pupil), vitreous opacities, vascular conjunctival abnormalities, glaucoma, and retinal angiopathy (18, 19).

Recently, retinal angiopathy has undergone investigation by Marques et al. through OCTA analysis in patients affected by hereditary ATTR. The authors associated the presence of a scalloped iris with a more advanced subclinical retinal angiopathy, observing an attenuated vascular density in the superficial and deep capillary plexuses (9).

Marta et al. have also studied choroidal involvement in these patients. They calculated the CVI, while Mano et al. developed a grading system to categorize the choroidal characteristics of patients with systemic amyloid severity (5–7).

Few data are present regarding ophthalmological abnormalities in a wild-type ATTR population (20). A recent cross-sectional study by Frizziero et al. evaluated ophthalmological findings in 17 ATTRwt, nine ATTRv, and two AL amyloidosis patients. Their analysis evaluated OCT and OCTA measurements of the macula and the optic disc, along with *in vivo* corneal confocal microscopy. A reduction of all OCTA vascular parameters at the macula and in the peripapillary plexus was registered in the ATTRwt group compared to controls, confirming vascular damage in this population. Compared to our study, considering that two different devices were used for the evaluation of OCTA vascular parameters, it is difficult to match the results.

A difference in the examined population is also present because Frizziero et al. evaluated not only ATTRwt patients, as in our study, but also ATTRv, carriers, and AL patients. Anyhow, we concur with Frizziero et al. in terms of reduced vascular OCTA indices.

Frizziero et al. also registered lower visual acuity in ATTRwt patients compared to the other groups, whereas this finding is not present in our study. The population included by Frizziero et al. was probably more severely affected by the disease and more aged compared to ours, and this issue could justify the discrepancy in the functional evaluation.

Furthermore, we recorded a significant reduction in both choroidal thickness and the CVI, suggesting that primarily the choroid and secondarily the retinal microvasculature are involved in the disease.

Utilizing a multimodal imaging analysis, we were able to establish correlations between the data collected for retinal angiopathy (vascular density at the levels of the superficial, deep, and choriocapillary plexuses) and the data assessed for choroidal condition evaluation (the CVI and CT).

In our analysis, the vascular density (VD) across the five ETDRS sectors within the superficial, deep, and choriocapillary plexuses exhibited a notable decrease among ATTRwt patients compared to the control group. This reduction was mirrored by CT changes and the CVI. These observations suggest a compromised blood supply to the entire macular region, affecting both the retinal microvasculature and the choroid. This implies a balanced involvement of the retina and choroid in this context.

To conduct a more precise analysis, we meticulously examined the macular area, assessing data from the five ETDRS macular sectors and individually segmenting the SCP, DCP, and choriocapillaris (CC).

We deemed this metric a pertinent measurement of choriocapillaris vascular density (VD) due to the heightened vulnerability of choroidal tissue in patients with systemic disorders, owing to its hemodynamic characteristics. As a result, compromise in this regard is likely to occur even in the absence of clinically detectable symptoms. Nonetheless, despite these structural alterations, there was no apparent visual deterioration. Consequently, we consider that these findings represent subclinical features. A thorough exploration and clarification of the underlying mechanisms responsible for vascular complications in the retina and choroid of ATTR patients is imperative. Establishing potential connections between localized ocular manifestations and systemic vascular processes holds the promise of valuable insights into the disease's pathophysiology, offering a basis for more effective diagnostic and therapeutic strategies. Our upcoming objective is to establish a correlation between increased arterial stiffness—common among these patients—and retinal and choroidal VD (21).

Today, new therapeutic options can be offered to these patients. tafamidis, a TTR stabilizer that has recently shown effectiveness in cardiac forms of both ATTRv and ATTRwt, is now available for clinical use (22). Moreover, drugs designed to silence the TTR messenger RNA will soon become available for treatment. All these emerging therapeutic possibilities for ATTR further emphasize the importance of early diagnosis for ATTRv and ATTRwt diseases.

Hence, we opted to perform a sub-analysis within the ATTRwt group to assess potential variations in VD or CVI indices between patients receiving tafamidis and those without therapy. No statistically significant differences were observed between the two groups. Given that the duration of tafamidis therapy in the studied population ranged from 1 to 7 months, this relatively short timeframe, coupled with heterogeneous data, might not provide adequate grounds to evaluate distinctions between the groups. Actually, the effectiveness of tafamidis in the vitreous may vary, and there may be concerns about its concentration reaching therapeutic levels. Oligonucleotide therapies have also shown promise in the treatment of ATTR (23). The potential of using intravitreal oligonucleotides in ATTRwt amyloidosis might be to specifically inhibit the production of wild-type transthyretin protein in the eye. By targeting the expression of transthyretin mRNA, oligonucleotides can potentially reduce the production of the protein and prevent its deposition in the vitreous humor and other ocular tissues.

We acknowledge certain limitations in our study. First, the smaller number of healthy subjects compared to ATTRwt patients results in an imbalance in sample sizes between the two groups. This disparity is also evident in the sub-analysis, where the count of patients without therapy is considerably lower than that receiving therapy. Therefore, a larger cohort of healthy subjects and untreated patients would likely enhance the study's statistical power. Second, while the study excluded patients with high myopia, it is important to note that axial length was not assessed in this subgroup, potentially introducing bias into the recorded CVI values (23–25). Finally, we did not track the longitudinal progression of ATTRwt patients, and thus, we lack information regarding whether the observed microvascular impairment is a persistent condition or subject to changes over time.

This study boasts several strengths. First, it delves into the analysis of patients afflicted by systemic ATTRwt disease, a group that has been poorly evaluated. Additionally, it conducts a comprehensive examination of each ETDRS subfield's VD and extends the analysis to the choriocapillaris level. Moreover, the study offers a contextual assessment of both retinal and choroidal microvasculature, enriching the scope of the investigation.

## Conclusion

Our findings support the hypothesis of compromised choroidal and retinal microvasculature in these patients, suggesting the potential utility of these parameters as biomarkers.

This evidence underscores the potential of OCTA and CVI indices as non-invasive bioimaging biomarkers for treatment monitoring and their possible correlation with systemic parameters. Our future study aims to incorporate a longitudinal assessment of OCTA and CVI indices in therapy patients to track changes over time.

## Data availability statement

The raw data supporting the conclusions of this article will be made available by the authors, without undue reservation.

## Ethics statement

Written informed consent was obtained from the individuals for the publication of any potentially identifiable images or data included in this article.

## Author contributions

FC: conceptualization and writing. MR and GL: original draft. FT and GP: data curation. GC: formal and statistical analysis. MD and MG: methodology. FN: review and editing. MC: genetic testing. FD: validation. FV: visualization and methodology. CC: review and validation. All authors contributed to the article and approved the submitted version.
